# Picosecond optospintronic tunnel junctions

**DOI:** 10.1073/pnas.2204732119

**Published:** 2022-06-06

**Authors:** Luding Wang, Houyi Cheng, Pingzhi Li, Youri L. W. van Hees, Yang Liu, Kaihua Cao, Reinoud Lavrijsen, Xiaoyang Lin, Bert Koopmans, Weisheng Zhao

**Affiliations:** ^a^Fert Beijing Institute, MIIT Key Laboratory of Spintronics, School of Integrated Circuit Science and Engineering, Beihang University, 100191 Beijing, China;; ^b^Department of Applied Physics, Institute for Photonic Integration, Eindhoven University of Technology, 5600 MB Eindhoven, The Netherlands

## Abstract

Perpendicular magnetic tunnel junctions (p-MTJs), as building blocks of spintronic devices, offer substantial potential for next-generation nonvolatile memory applications. However, their performance is fundamentally hindered by a subnanosecond speed limitation, due to spin-polarized-current-based mechanisms. Here, we report an optospintronic tunnel junction (OTJ) device with a picosecond switching speed, ultralow power, high magnetoresistance ratio, high thermal stability, and nonvolatility. This device incorporates an all-optically switchable Gd/Co bilayer coupled to a CoFeB/MgO-based p-MTJ, by subtle tuning of Ruderman–Kittel–Kasuya–Yosida interaction. An all-optical “writing” of the OTJ within 10 ps is experimentally demonstrated by time-resolved measurements. The device shows a reliable resistance “readout” with a relatively high tunnel magnetoresistance of 34.7%, as well as promising scaling toward the nanoscale with ultralow power consumption (<100 fJ for a 50-nm-sized bit). Our proof-of-concept demonstration of OTJ might ultimately pave the way toward a new category of integrated spintronic–photonic memory devices.

## Significance

Spintronic devices have become promising candidates for next-generation memory architecture. However, state-of-the-art devices, such as perpendicular magnetic tunnel junctions (MTJs), are still fundamentally constrained by a subnanosecond speed limitation, which has remained a long-lasting scientific obstacle in the ultrafast spintronics field. The highlight of our work is the demonstration of an optospintronic tunnel junction, an all-optical MTJ device which emerges as a new category of integrated photonic–spintronic memory. We demonstrate 1) laser-induced deterministic and efficient writing by an all-optical approach and electrical readout by tunnel magnetoresistance, 2) writing speed within 10 ps, demonstrated by femtosecond-resolved measurements, and 3) integration with state-of-the-art MTJ performance and a complementary metal–oxide–semiconductor-compatible fabrication progress.

For decades, CoFeB/MgO-based perpendicular magnetic tunnel junctions (p-MTJs) have become the building blocks of spintronic devices, offering a promising candidate for next-generation nonvolatile memory architectures (1–3). As to the switching mechanisms of p-MTJs, magnetic field-induced and spin-polarized current-induced switching schemes (4–9) have been widely employed, including spin transfer torque and spin orbit torque. Unfortunately, limited by the spin precession process, the switching speed of these p-MTJs is still around subnanosecond while requiring extremely high current densities (10–12), the improvement of which remains a long-existing challenge in the field of ultrafast spintronics and femtomagnetism.

To address this issue, integrating a femtosecond (fs) laser, i.e., the fastest stimulus commercially available, has been conceptually considered as a competitive route toward ultrafast nonvolatile memory (13, 14). About one decade ago, single-pulse all-optical switching (AOS) was discovered in ferrimagnetic systems (15, 16), where the magnetization could be fully reversed using a single fs laser pulse. It features ultrafast (<20 ps) and energy-efficient (<100 fJ for a ∼50-nm-sized bit) data writing, showing high potential toward integrating with spintronic memories. In this regard, by integrating AOS with a high-performance p-MTJ, an all-optically addressable MTJ device has been envisioned, which would be highly promising to enable a unique category of integrated photonic–spintronic memory devices with picosecond switching speed and low energy consumption (13, 14). It also enables a direct conversion from the photonic domain to the magnetic domain, without the energy-costly electronic steps. However, key scientific issues related to physical mechanisms, material exploration, and device fabrication still need to be solved.

Fundamentally, implementation of such an opto-spintronic device involves physical effects including single-pulse helicity-independent AOS (AO-HIS) (15–17) to ensure a picosecond data writing, as well as an appreciable tunnel magnetoresistance ratio (TMR) to enable a reliable resistance readout (4, 6, 9). In addition, a strong perpendicular magnetic anisotropy (PMA) is highly required (5, 9) to increase data storage density and improve thermal stability. Incorporation and optimization of these physical effects to realize the desired device functionalities further raise requirements on optospintronic tunnel junction (OTJ) stack material design. The OTJ stack includes an optically switchable free layer (FL) to enable fs laser-induced switching, a CoFeB/MgO-based MTJ structure to ensure a high TMR, a synthetic antiferromagnetic (SAF) reference layer (RL) used as a bottom-pinned structure, as well as a transparent top electrode to enable optical access. Among them, the FL structure is expected to be compatible with a robust AO-HIS, a high TMR, and a strong PMA, simultaneously.

In this regard, a composite FL incorporating an all-optically switchable layer coupled to a CoFeB/MgO structure has become a mainstream technological route (13, 18, 19). Among these attempts, Avilés-Félix et al. reported single-pulse AOS of an MTJ electrode using Co/Tb multilayers (19). However, scientific and engineering issues, such as toggle writing of the device and its time-resolved switching dynamics, as well as the incorporation of a bottom-pinned RL structure, still need to be solved before practical applications.

As to the device functionalities, a robust single-pulse laser-induced TMR toggle switching, as well as a high thermal stability at the nanoscale with nonvolatility, should be satisfied simultaneously. Among the attempts, Chen et al. employed an all-optically switchable GdFeCo layer as the single FL to fabricate an MTJ (20), resulting in a relatively low TMR ratio of 0.6% and weak PMA. Moreover, all the data shown in ref. 20 used an averaging time of 100 ms and no time-resolved data were provided. Therefore, the switching speed and the time it takes to recover full magnetization have not been resolved, and in fact it remained unclear whether the full FL switched simultaneously. Therefore, to fully explore its great potential toward next-generation memory applications, experimental demonstration and characterization of such a fully-functional optospintronics device are highly desired.

In this work, we design and experimentally demonstrate a proof-of-concept OTJ device with an ultrafast switching speed (<10 ps), sufficiently high TMR ratio (34.7%), low threshold fluence (3.1 mJ/cm^2^), and high thermal stability and nonvolatility. By using Ruderman–Kittel–Kasuya–Yosida (RKKY) interaction, a composite FL structure, with an all-optically switchable ferrimagnetic Co/Gd bilayer coupled to a ferromagnetic CoFeB layer is developed. Such an interlayer exchange interaction through an atom-thick Ta spacer realizes the simultaneous AOS of the composite FL with a high PMA and TMR effect. The proposed OTJ exhibits efficient all-optical toggle writing, as well as a reliable electrical TMR readout in real time even for single-shot switching using a 100-fs laser pulse. Moreover, the switching speed of the OTJ within 10 ps is confirmed by time-resolved magneto-optical measurements, with a promising scaling toward nanoscale devices.

## Results

### OTJ Film Structure and PMA Characterization.

**Fig. 1.**
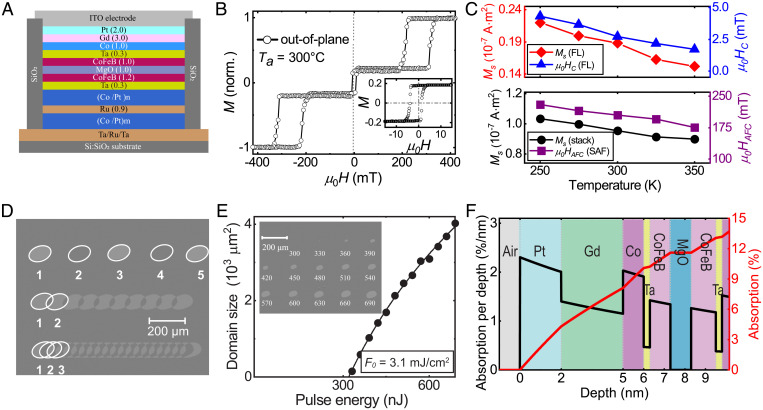
OTJ structure, magnetic characteristics, and efficient AOS of the stack. (*A*) Schematic structure of the proposed OTJ device. Ta/Ru/Ta and ITO are the bottom and top electrode, respectively. [Co/Pt]_m/n_ multilayer-based SAF is used as the RL and MgO as the tunnel barrier. The composite FL consists of the top CoFeB layer and the Co/Gd bilayer, which are RKKY-coupled through an atom-thick Ta spacer. (*B*) Out-of-plane hysteresis loop of the OTJ stack after postannealing at 300 °C measured by a VSM-SQUID. The major loop is measured by sweeping the out-of-plane magnetic field from −450 mT to 450 mT, which results in switching of FL and the SAF layer. The square hysteresis loops with 100% remanence indicate a well-defined PMA in both FL and RL. The minor loop of the OTJ stack shown in the inset indicates that two parts of the FLs switch simultaneously due to the strong RKKY coupling. (*C*) Temperature dependence of the magnetic properties of the OMTJ stack. (*Bottom*) Temperature dependence of the saturation magnetization ($M_{s}$) of the complete stack (left, black dots) and antiferromagnetic coupling field ($\mu_{0}H_{\mathrm{AFC}}$) (right, purple squares) ranging from 250 K to 350 K. (*Top*) Temperature dependence of $M_{s}$ of the FL (left, red diamond) and their coercive field ($\mu_{0}H_{c}$) (right, blue triangles). (*D*) Deterministic single-pulse AOS measured by Kerr microscopy. Row 1: Single-pulse AOS measurement by subsequent fs laser pulses. The numbers correspond to the number of pulses that the region is exposed to. Rows 2 and 3: AOS measurements by laser pulse train at a repetition rate of 2.5 Hz and 5 Hz, respectively, leading to partly overlapping pulses. (*E*) Laser-pulse energy dependence of the AOS domain size, showing the threshold laser energy ($P_{0}$) of 330 nJ and an increasing domain size with higher laser energies. The threshold fluence ($F_{0}$) of the OTJ stack is calculated to be 3.1 mJ/cm^2^. (*Inset*) Kerr microscopy of the measurement performed on the OTJ stack. The numbers correspond to the laser energy of each spot in units of nanojoules. (*F*) Calculation of optical absorption profile of the OMTJ stack used in this work. The full stack structure as well as the thermally oxidized Si:SiO_2_ substrate are included in the calculation. Optical absorption per depth (percent per nanometer) vs. layer depth.

As illustrated in Fig. 1*A*, the designed OTJ stack is composed of, from the substrate side upward, Ta (3)/Ru (20)/Ta (0.7)/[Co/Pt]_m_ (9.8)/Ru (0.8)/[Co/Pt]_n_ (3.6)/Ta (0.3)/CoFeB (1.2)/MgO/CoFeB (1)/Ta (0.3)/Co (1)/Gd (3)/Pt (2) (numbers in parentheses denote the layer nominal thicknesses in nanometers, and the subscripts are the repeated numbers), which is deposited by direct current (DC) and radio frequency (RF) magnetron sputtering (see *Materials and Methods*). In this configuration, Ta/Ru/Ta is the bottom electrode, and the combination of the [Co/Pt]_m/n_ multilayers with the bottom CoFeB serves as the SAF RL. Such a design is used to fix the magnetization direction of the CoFeB, which is crucial for practical device applications, such as magnetoresistive random-access memories. The MgO layer is the tunnel barrier. The 2-nm-thick top Pt layer is employed as a protective layer to prevent oxidation to the MTJ stack.

As to the composite FL, the top CoFeB/MgO layer and the all-optically switchable Co/Gd bilayer are coupled through an 0.3-nm-thick Ta spacer. Such a design satisfies a high TMR, a high PMA, and an ultrafast AOS at the same time. Specifically, we recently demonstrated single-pulse AOS of ferrimagnetic Co/Gd bilayers, highly promising toward future ultrahigh-density spintronic memories (17, 21–23). Apart from the efficient and robust single-pulse AOS, they show extended flexibility on interface engineering, such as high domain wall (DW) velocity, and inherent built-in interfacial Dzyaloshinskii–Moriya interaction, which are indispensable components for future ultrahigh-density spintronic memories (24–26). The Ta layer is used to ensure an effective RKKY coupling, where a moderate coupling strength is adopted to guarantee an all-optical toggle switching of the composite FL. Such an atom-thick Ta spacer is widely regarded as a mainstream route to enable a strong interlayer coupling in the MTJ technology (6, 27, 28).

After deposition, the stacks are thus subjected to thermal annealing at 300 °C in vacuum for an hour, which is a relatively standard process in CoFeB/MgO-based MTJ fabrication to obtain a decent TMR (6, 9, 27, 28). Specifically, it is used to enhance the crystalline quality of the MgO barrier and the bcc (body-centered cubic) texture of the CoFeB, thus a higher TMR can be expected. Although alloyed AOS materials are extremely limited by their composition and high annealing temperature, in our recent study (29) we showed that the AOS efficiency of a Gd/Co bilayer is considerably enhanced after annealing up to 300 °C. This offers a fully compatible fabrication process with p-MTJ integration.

Fig. 1*B* shows the out-of-plane magnetization versus magnetic field (*M-H*) hysteresis loop of the OTJ stack using a vibrating sample magnetometer–superconducting quantum interference device (VSM-SQUID) at room temperature (see *Materials and Methods*). The film exhibits the presence of strong PMA in both the FL and the RL, indicated by the 100% remanence and squareness of the hysteresis loops, which ensures high thermal stability and nonvolatility. According to the steep minor loop (Fig. 1*B*, *Inset*), the coercive field of the FL reaches 4 mT, and two parts of the FLs switch simultaneously, which is paramount for OTJ performance.

Note that a relatively small shift of the hysteresis loop indicates a significant reduction of stray field due to the Co/Pt multilayered-based SAF structure. Such a pinned layer design is essential for our OTJ, because a large stray field would hinder AOS in the opposite direction, losing toggle switching characteristics. In addition, the *M-H* hysteresis loop of the OTJ ranging from 250 K to 350 K is measured, as shown in *SI Appendix*. Fig. 1*C* shows the temperature dependence of the magnetic properties, which are extracted from the *M-H* hysteresis loops. The left axis in the bottom panel shows the saturation magnetization ($M_{s}$) of the full stack (black dots), whereas the left axis in the top panel shows the $M_{s}$ of the FL (red diamonds). A clear temperature dependence of $M_{s}$ is observed, enhancing upon lower temperature. Note that although the Curie temperature of the bulk Gd is slightly lower than room temperature, it is enhanced at the Co interface due to the proximity-induced effect (17). The right axis of the bottom panel shows the temperature dependence of the antiferromagnetic coupling field ($\mu_{0}H_{\mathrm{AFC}}$) of the SAF layer (purple squares). Specifically, compared with $\mu_{0}H_{\mathrm{AFC}}$ = 175 mT at 350 K, it is enhanced to 230 mT at 250 K. The right axis of the top panel shows the coercive field ($\mu_{0}H_{c}$) of the FL (blue triangles), which is also enhanced from 1.7 mT to 4.3 mT, corresponding to a 153% increase. These results indicate a broad working temperature range of the OTJ stack for practical applications.

### Single-Pulse All-Optical Switching of the OTJ Film.

We first investigate the single-pulse AOS of the OTJ full stack before device patterning. In the measurements (see *Materials and Methods*), the sample is exposed to subsequent linearly polarized laser pulses, whereafter its magnetization response is measured using magneto-optical Kerr microscopy in a static state. As shown in Fig. 1*D*, five separate spots are excited by a different number of laser pulses (row 1). We observe that a homogeneous domain with an opposite magnetization direction is written for every odd number of laser pulse, whereas it toggles back for every even number of pulses. Then, the OTJ stack is exposed to a laser-pulse train with partly overlapping area (rows 2 and 3; see *Materials and Methods*). As to the regions exposed by a single or three overlapping pulses, a reversed domain is observed. In contrast, for the regions where only two pulses overlap, no net reversal is observed. Here, we also note that the DW at the overlapping regions stay intact, whereas the domains toggle upon every laser excitation. The observed results are consistent with the thermal AOS mechanism discussed in previous studies (17, 21), demonstrating a robust toggle AOS switching in the OTJ stack.

As to the ultralow power spintronic device, it is always essential to reduce the critical writing energy needed to switch a bit cell. Therefore, the threshold laser energy to write the OTJ stack is investigated, which is done by measuring the pulse energy dependence of the domain size (see *Materials and Methods*). As shown in Fig. 1*E*, a threshold laser energy ($P_{0}$) of 330 nJ is observed for a laser spot with a diameter of ∼50 μm. Above $P_{0}$, the laser-written domain size increases with higher laser energies. The threshold fluence (*F*_0_) describes the critical laser fluence needed for AOS, which is derived from the optically written domain size as a function of the laser pulse energy. By fitting the measurement data and assuming a Gaussian shape of the laser pulse (17), *F*_0_ = 3.1 mJ/cm^2^ is obtained, which is slightly lower than that for GdFeCo alloys (15, 16). Further reduction of *F*_0_ can be expected by decreasing the thickness of the ferromagnetic layer, as discussed in previous studies (17). Moreover, due to the thermal nature of the AOS, the energy for writing a sub-20-nm OTJ bit could scale down to a few tens of femtojoules using a plasmonic antenna (21, 30, 31), as already used in heat-assisted magnetic recording techniques, which is potentially competitive with state-of-the-art spintronic memories (1, 13).

To evaluate the feasibility of the AOS in the RKKY-coupled FL, we calculate the optical absorption of each layer in the OTJ stack using a transfer matrix scheme. The optical profile of the complete stack is shown in Fig. 1*F*. The optical absorption in this OTJ stack is calculated by inputting the layer-specific refractive index values at 700 nm. The left axis indicates the absorption per depth (percent per nanometer), as a function of layer depth. The absorption in the layer is then calculated by integrating over the thickness, as plotted in the right axis. One might expect that the relatively thick stack, with a Pt layer on top, would lead to a strong loss of optical energy, and thus negligible laser-induced phenomena in the OTJ. However, in our calculation, we observe that nearly 7.2% of the laser energy is absorbed by the composite FL layer with a configuration of Gd (3)/Co (1)/Ta (0.3)/CoFeB (1), of which 1.5% in the Co layer. We find that this value is significantly enhanced due to the Si:SiO_x_ substrate, which acts as a reflective layer (32). The absorption in the Co layer, which determines the heating of the electron temperature near the Co/Gd interface that seeds the AOS process, is rather similar to previous studies using simple Gd/Co bilayers (17, 22). This explains why the $F_{0}$ of the OTJ stack does not differ significantly from such a more conventional bilayer AOS system (17).

### OTJ Device Fabrication and TMR Measurement.

**Fig. 2.**
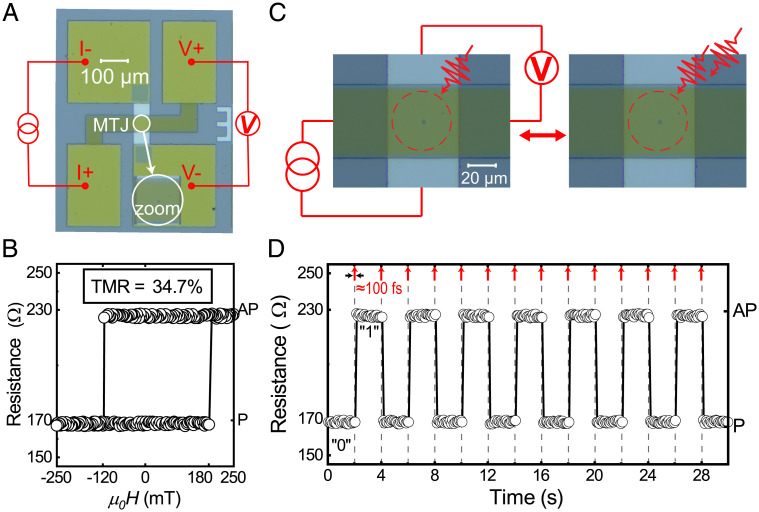
OTJ device functionalities: all-optical “writing” and electrical TMR readout. (*A*) Microscope image of the fabricated OTJ device with a 100-nm-thick transparent ITO top electrode, as well as four electrode pads to perform four-point TMR detection. (*Inset*) Zoom-in of the OTJ pillar. (*B*) *R-H* magnetoresistance loop measured by sweeping an out-of-plane magnetic field, showing a typical TMR ratio of 34.7%. (*C*) Schematic overview of the AOS “writing” of the OTJ device. A small current is applied through the OTJ, while the resulting TMR voltage is measured in real time. The OTJ pillar is excited by a train of linearly polarized laser pulses. (*D*) Typical TMR measurement as a function of time upon laser-pulse excitation. The resistance toggles between P and AP state upon every laser pulse excitation. No external field was applied during the measurements.

Circular pillars are patterned using multistep ultraviolet (UV) lithography and an Ar ion milling process (see *Materials and Methods*). Fig. 2*A* shows an optical microscope image of the fabricated OTJ device with a pillar diameter of 3 μm (see Fig. 2*A*, *Inset* for the zoom-in of the pillar) and four electrode pads. Note that the 100-nm-thick indium tin oxide (ITO) is employed as top electrode, which is crucial for our hybrid optospintronic device. Compared with the conventional Ti/Au electrode, the transparent ITO enables an efficient laser-pulse access, as well as a reliable electrical detection with a high signal-to-noise ratio (SNR). The resistance versus magnetic field (*R-H*) loop is measured by sweeping an out-of-plane magnetic field, as shown in Fig. 2*B*. A clear bistable tunnel magnetoresistance (TMR) is observed, with $R_{AP}$ = 227 Ω (AP: antiparallel) and $R_{P}$ = 169 Ω (P: parallel), respectively. The RKKY-coupled FL is found to switch as a single unit, as indicated by steep resistance transition events at +190 mT and −115 mT, respectively. A typical TMR ratio $\left(R_{Ap}-R_{P}\right)/R_{P}$ = 34.7% is obtained for our proof-of-concept OTJ device after postannealing, which could be further enhanced by optimizing the stack design but goes beyond the aim of the present work. In addition, the SNR in the *R-H* measurement indicates an improved ITO quality as well as the interface between the ITO and the OTJ pillar (20), which is achieved by tuning the ITO process parameter during electron beam evaporation.

### All-Optical “Writing” of the OTJ Device.

Next, we investigate the electrical read-out performance of OTJ. As sketched in Fig. 2*C*, the programming of the OTJ is demonstrated using subsequent laser pulses, whereas the read operation is realized by an electrical TMR measurement in real time with down to subnanosecond time resolution (see *Materials and Methods*). The complete measurement is performed without any external magnetic field. As shown in Fig. 2*D*, the TMR of the OTJ device toggles deterministically between the AP and the P state at the same frequency as the incoming laser pulses. In other words, binary programming a “1”/“0” of the OTJ is realized for every odd/even number of pulses, which is a basic writing operation for memory devices. The repeatability of the operation is further verified by a 100% success rate up to millions of repeated switches. Moreover, the magnetoresistance values measured by AOS are equal to the ones in the *R-H* loop (Fig. 2*B*), unambiguously demonstrating a complete reversal of the composite FL in the bottom-pinned OTJ. We stress that this robust AOS is not trivial, since for our synthetic FL with a more complex structure the toggle AOS could have been hindered by the stray field provided by the RL (∼30 mT). Thus, the tendency for AOS of our entire FL is strong enough to overcome such bias field, and a toggle TMR operation is clearly observed.

**Fig. 3.**
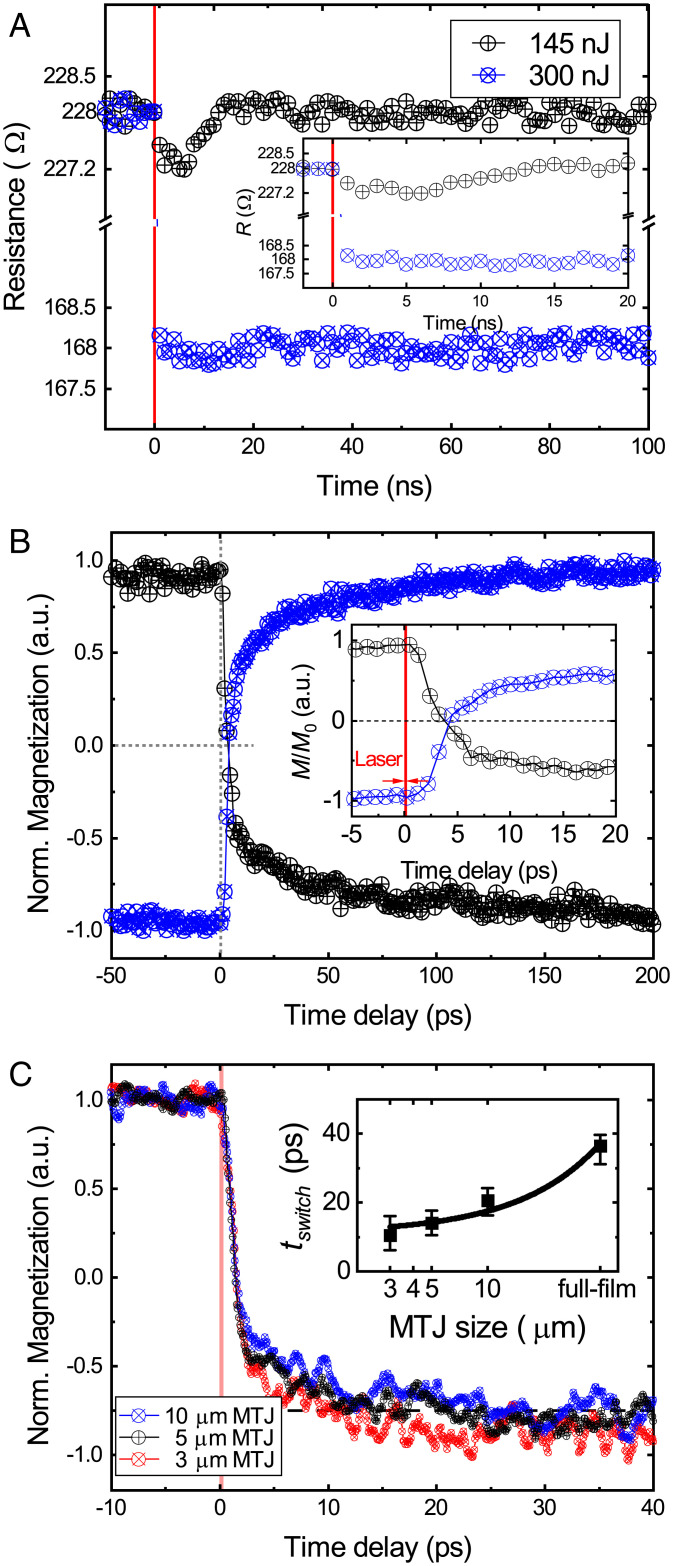
Picosecond speed demonstration of the OTJ by time-resolved MOKE measurements. (*A*) Typical time-resolved TMR measurement result using a fast sampling oscilloscope with a subnanosecond resolution. In the measurement, the OTJ is excited by subsequent laser pulses, whereas the TMR is measured in real time by a fast sampling oscilloscope. In case of the laser energy of 300 nJ, we observe a clear TMR switching with the speed faster than 1 ns. However, in case of the laser energy of 145 nJ, only a thermal relaxation with a time scale of 10 ns is observed. This results clearly indicate that the switching speed of OTJ is beyond the nanosecond regime. (*B*) TR-MOKE measurements performed on the OTJ full stack. (*Inset*) Zoom-in of the first 20-ps time scale. The switching speed is confirmed by magnetization reversal within 5 ps. (*C*) TR-MOKE measurements performed on the OTJ with pillar diameters of 10 μm, 5 μm, and 3 μm, respectively. The results show that the ultrafast AOS speed is unimpeded in patterned devices, as proven by the magnetization reversal still within the picosecond time scale. (*Inset*) The time needed for 75% magnetization reversal ($t_{\mathrm{switch}}$) as a function of OTJ pillar size. The results may indicate a scaling dependence of $t_{\mathrm{switch}}$, i.e., compared to $t_{\mathrm{switch}}$ ∼ 40 ps for the full-sheet stack, it reduces to 10 to 25 ps for the OTJ device.

To further address the potential of our OTJ for high-data-rate electrical readout, we investigated the time-resolved TMR upon single-pulse laser excitation. Here, the OTJ is again exposed to subsequent laser pulses with a pulse width of 100 fs, whereas a fast-sampling oscilloscope with a subnanosecond resolution is employed to enable a time-resolved measurement (see *Materials and Methods*). As a typical result shown in Fig. 3*A*, in case of a laser energy of 300 nJ, a robust switching faster than 1 ns is observed. However, as expected, in case of a laser energy of 145 nJ, i.e., below the threshold for switching, only an ultrafast thermal demagnetization followed by a remagnetization during 10 ns upon cooling down is observed. Finally, we stress that the design rules of such OTJs are not limited to Gd/Co bilayers. Under proper material design to maintain ultrafast AOS, high TMR, as well as high thermal stability simultaneously after postannealing, other emerging ferrimagnetic AOS systems may also hold great potential for further optimized device performance (13, 14, 18).

### Femtosecond-Time-Resolved Measurements of the OTJ Switching.

Finally, we demonstrate the picosecond switching speed of the OTJ, which is a key characteristic for the next-generation ultrafast devices. Previous studies have reported tens-of-picosecond AOS in some ferrimagnetic systems (15, 18, 33). However, no time-resolved AOS dynamics have been evaluated in a complete MTJ device with a RKKY-coupled FL yet, and questions could be raised regarding the simultaneous switching of this composite FL, the speed at which this switch would occur, and the role of the relatively large stray field (∼30 mT) due to the SAF RL therein. Additionally, note that the underlying mechanism of the Gd/Co bilayer is different from that of a Gd-based alloy (22). Therefore, time-resolved magneto-optical Kerr (TR-MOKE) measurements are performed (see *Materials and Methods*) to clarify such concerns. As illustrated in Fig. 3*B*, the laser-induced magnetization dynamics of the OTJ stack shows typical characteristics that are consistent with previous studies on time-resolved AOS studies (15, 31, 33). Briefly, in the first several-picosecond time scale, a rapid demagnetization is caused by the ultrafast laser heating. Afterward, magnetization reversal takes place within 20 ps, due to the distinct demagnetization times (15) between the antiferromagnetically coupled Co and Gd sublattices, which is driven by the angular momentum transfer mediated by exchange scattering (22). Finally, a reversed magnetization orientation indicates its settling to a new thermal equilibrium. More importantly, we observe the zero-crossing point (see Fig. 3*B*, *Inset* for zoom-in) occurring at several picoseconds, and a full reversal to the saturated reversed state within tens of picoseconds. These results confirm that the CoFeB layer follows the Co/Gd quasi-instantaneously on a picosecond time scale and highlight the ultrafast feature of the OTJ device.

After we have shown that the composite FL ensures single-pulse AOS with high PMA and thermal stability, we finally demonstrate a proper scaling behavior between the ultrafast switching speed and the patterned device size. TR-MOKE measurements on devices with pillar diameters of 10 μm, 5 μm, and 3 μm are performed, respectively, as shown in Fig. 3*C*. Note that the relatively low SNR, compared to Fig. 3*B*, is attributed to the OTJ pillar size’s being smaller than the laser spot size (typically 20 μm), reducing the total magnetic signal collected by the Kerr effect. We observe that the picosecond AOS speed is preserved upon scaling down to 3 μm, as proved by the magnetization reversal within 5 ps. For application purposes, we define the switching speed of the OTJ as the time ($t_{\mathrm{switch}}$) needed to reach 75% opposite magnetization state, which is chosen because of a sufficient read margin for binary electronics chips (31). As plotted in Fig. 3*C*, *Inset*, $t_{\mathrm{switch}}$ of all the measured OTJ devices is in the range of tens of picoseconds. In addition, our results may even indicate a scaling dependence of $t_{\mathrm{switch}}$; more specifically, a faster switching speed is observed for reduced dimensions. After considering the potential influence of the limited SNR, the trend in our results is in reasonable agreement with recent studies using GdCo nanodots (31). Especially, compared to $t_{\mathrm{switch}}$ ∼ 40 ps for the full-sheet stack, it reduces to 10 to 25 ps for microsized OTJ devices (Fig. 3*C*, *Inset*). We conjuncture that the nonuniform heat diffusion to the ambient, which causes the magnetization to settle to a new thermal equilibrium faster, may play a crucial role in this observed size dependence. However, other laser-induced magnetization phenomena, such as spin–lattice coupling and energy transfer rates as speculated in previous studies (31), may not be fully ruled out.

## Discussion

In conclusion, we report an integrated spintronic–photonic OTJ device with picosecond switching speed, ultralow power, sufficiently high TMR, and nonvolatility. The device incorporates an all-optically switchable ferrimagnetic Co/Gd bilayer that is RKKY-coupled to a CoFeB/MgO-based p-MTJ to ensure robust AOS, high TMR, and PMA simultaneously. The all-optical toggle switching of the OTJ within 10 ps is unambiguously demonstrated, and proper scaling at least toward micrometer-sized devices is shown. A considerably high TMR of 34.7% for electrical readout and a low threshold laser fluence below 100 fJ for a ∼50-nm-sized bit for efficient optical writing are obtained. Therefore, the proof-of-concept OTJ device might pave the way toward an entirely new category of integrated photonic–spintronic memory devices. By enabling a nonvolatile memory of photonic information into the magnetic domain, the proposed OTJ would also extend the inherent advantages of photonics such as data transfer and processing.

## Materials and Methods

### Sample Deposition.

The OTJ stacks used in this work were deposited using DC and RF magnetron sputtering (AJA International Physical Vapor Deposition) at room temperature, which were deposited on a thermally oxidized Si (001) substrate at a base pressure in the deposition chamber of 10^−9^ mbar without an external magnetic field. The CoFeB target composition was Co_20_Fe_60_B_20_ (in atomic percent), with a deposition rate of 3 min/nm at Ar pressure of 8 × 10^−4^ mbar. The deposition rate for MgO was 5.5 min/nm at Ar pressure of 8 × 10^−4^ mbar. After deposition, the stacks were annealed in vacuum (with a base pressure of 10^−9^ mbar) at 300 °C for 1 h without an external magnetic field.

### Device Fabrication.

Microsized OTJ pillars were patterned by using a standard UV optical lithography in combination with argon ion milling process, at the center of Ta/Ru/Ta bottom electrode. The diameters of the OTJ pillars were 10, 5, 4, and 3 μm, respectively. The samples were then covered with SiO_2_ insulation by electron beam evaporation (EBV) and a lift-off procedure. Subsequently, the 100-nm-thick transparent ITO was deposited as the top electrode, also by EBV and a standard lift-off procedure. The quality of the ITO deposition process is essential for the OTJ device, leading to an efficient laser-pulse access and a reliable electrical detection as well.

### Magnetic Properties Characterization.

The magnetic characteristics of the full-sheet OTJ stack were investigated at 250 to 300 K (Fig. 1*B* and *SI Appendix*, Fig. 1), using a VSM-SQUID, under an out-of-plane magnetic field ranging from ±450 mT. The minor loop refers to the hysteresis loop of the FL. This is measured by sweeping the out-of-plane magnetic field ranging from −15 mT to 15 mT, in other words, only switching the FL of the MTJ.

The tunnel magnetoresistance of the OTJ was characterized at room temperature by a conventional four-point TMR measurement under an out-of-plane magnetic field in the range of ±200 mT. To measure the TMR signal, a small current (100 μA) was sent through the OTJ device, and the resulting magnetoresistance was measured using a lock-in amplifier.

### Deterministic AOS Measurements.

The response of magnetization in the OTJ stack upon subsequent fs laser pulses (Spirit-NOPA; Spectra Physics) were investigated. The laser pulse was linearly polarized, with a pulse duration of ∼100 fs at sample position, a central wavelength of 700 nm, a spot radius (1/e Gaussian pulse) typically of 25 μm, and a base repetition rate of 500 kHz. By using a pulse picker and a mechanical shutter, individual laser pulses could be picked out. In the single-pulse AOS measurements (Fig. 1*D*, row 1), which were performed at room temperature, the magnetization was first saturated by an external field. Afterward, the field was turned off and the stack was exposed to subsequent laser pulses. The numbers labeled in the figure correspond to the laser-pulse numbers of each spot. Then, the OTJ stack was exposed to laser-pulse trains with partly overlapping areas. The laser-pulse train was set at a velocity of 0.2 mm/s, with a repetition rate of 2.5 Hz (row 2) and 5 Hz (row 3).

The responses of the magnetization after laser-pulse excitation were measured in steady state using magneto-optical Kerr microscopy, where light and dark regions were corresponding to up and down magnetization direction. As to the Kerr microscopy images, a differential technique was used to enhance the magnetic contrast. Specifically, a “background” image was captured in the magnetization saturation state. This “background” was then subtracted from the subsequent Kerr images after laser-pulse excitation. The scale bars in the Kerr images are 200 μm.

To determine the AOS threshold fluence of the OTJ stack, the OTJ stack was exposed to single laser pulses with different laser energies ($E_{P}$) as indicated in Fig. 1*E*, *Inset* (in units of nanojoules), whereafter the laser-pulse energy dependence of the AOS domain size was measured by Kerr microscopy (see Fig. 1*E*, *Inset* for the Kerr microscopy image). By assuming a Gaussian energy profile of the laser pulse, the AOS threshold fluence could then be determined.

### AOS of the OTJ Device.

To investigate the all-optical programming switching of the device, the fabricated OTJs were excited by subsequent linearly polarized fs laser pulses, with the same laser configuration described above. The laser-pulse train was set at a relatively low repetition rate of 0.5 Hz to identify each single pulse. Meanwhile, as to the read operation, we measured its real-time electrical readout upon laser-pulse excitation, using a four-point TMR measurement and a fast-sampling oscilloscope, respectively. The complete measurements were performed without external magnetic field.

### TR-MOKE Measurements.

TR-MOKE measurements were performed using a typical pump-probe configuration at a repetition rate of 100 kHz. In the measurements, the sample was first exposed by a pump pulse with a duration of 100 fs, a spot size of typically 35 μm, and a laser-pulse energy of typically 600 nJ to write an AOS domain. Meanwhile, a probe pulse, which arrived at the sample with a different time delay and much lower laser energy, measures the time evolution of the magnetization via the magneto-optic Kerr effect. The probe spot size was typically 12 μm for successful detection for OTJ pillars, integrating the magnetic signal over the full OTJ pillar element. Due to the toggle switching behavior of the OTJ, a constant “reset” magnetic field was applied during the measurement to reset the magnetization between pump pulses. This method, as well as the data processing method, is consistent with other time-resolved AOS studies and can be found in refs. 31 and 34.

## Supplementary Material

Supplementary File

## Data Availability

All study data are included in the article and/or *SI Appendix*.
