# Supplementary material for: Picosecond optospintronic tunnel junctions
Source: Proc Natl Acad Sci U S A. 2022 Jun 6;119(24):e2204732119. doi: 10.1073/pnas.2204732119 (PMC9214493; doi:10.1073/pnas.2204732119)
Supplement: Supplementary File [file pnas.2204732119.sapp.pdf]

PNAS
www.pnas.org

Supplementary Information for
Picosecond Optospintronic Tunnel Junctions

Luding Wang, Houyi Cheng, Pingzhi Li, Youri L. W. van Hees, Yang Liu, Kaihua Cao, Reinoud Lavrijsen, Xiaoyang Lin*, Bert Koopmans, and Weisheng Zhao*

*corresponding authors: Xiaoyang Lin, Weisheng Zhao
Email: XYLin@buaa.edu.cn; weisheng.zhao@buaa.edu.cn

This PDF file includes:

Supplementary text
Figures S1

Supplementary Information Text

Temperature dependence of magnetic characteristics of the OTJ stack

To investigate the temperature dependence of the magnetic properties of the OMTJ stack, the magnetic moment versus external magnetic field (M-H) loops are measured at different temperature using the full-sheet sample, with the stack structure as shown in the main text. To compare the magnetic properties at different temperature, we use only one sample with a square shape for all the measurements. The diamagnetic background has been subtracted from the data at each temperature.

Supplementary Figure 1 shows the out-of-plane hysteresis loops measured by a VSM-SQUID (vibrating sample magnetometer - superconducting quantum interference device), at 250 K, 300 K, and 350 K, respectively. This temperature range is chosen because of the operation temperature (~ -20℃ - 80℃) for practical electronic device applications.

We observe that the strong RKKY coupling between the Gd/Co bilayer and the top CoFeB layer is withstood in such temperature range, which is evidenced by the sharp reversal process of the free layer (FL). The strong RKKY coupling in the free layer is crucial for our OMTJ performance. In addition, perpendicular magnetic anisotropy (PMA) exhibits in both of the FL and the reference layer (RL), as indicated by the squareness and high remanence of the hysteresis loops. These results indicate the feasibility of the OMTJ stack in a practical environment, highly promising towards future chip applications.

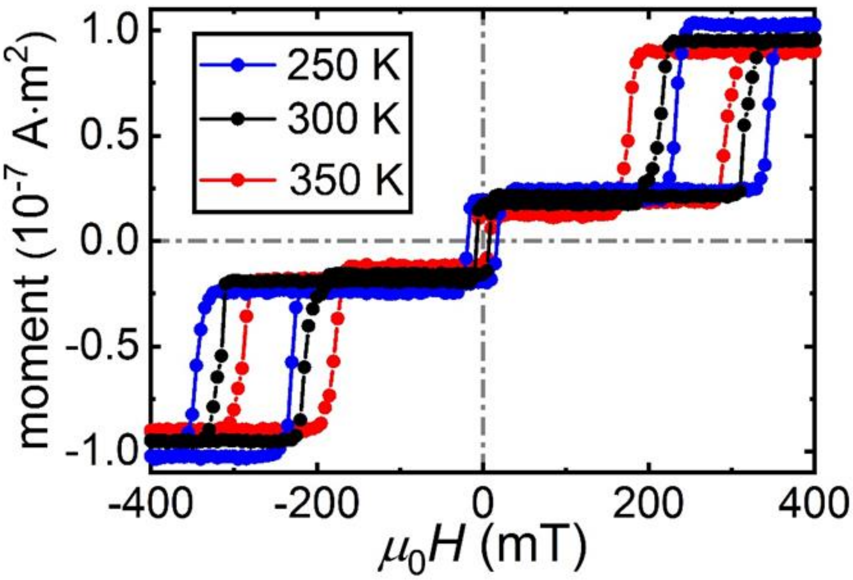

Fig. S1. Out-of-plane hysteresis loops of the OMTJ stack at different temperature. Magnetic moment versus an external perpendicular magnetic field (*M*-*H*) loops, measured at 250 K, 300 K, 350 K, respectively.
